# Fluidic Flow Enhances the Differentiation of Placental Trophoblast-Like 3D Tissue from hiPSCs in a Perfused Macrofluidic Device

**DOI:** 10.3389/fbioe.2022.907104

**Published:** 2022-06-30

**Authors:** Pengwei Deng, Kangli Cui, Yang Shi, Yujuan Zhu, Yaqing Wang, Xiaoguang Shao, Jianhua Qin

**Affiliations:** ^1^ Division of Biotechnology, Dalian Institute of Chemical Physics, Chinese Academy of Sciences, Dalian, China; ^2^ Division of Biotechnology, Dalian Institute of Chemical Physics, University of Chinese Academy of Sciences, Beijing, China; ^3^ Dalian Key Laboratory of Reproduction and Mother-child Genetics, Dalian Women and Children’s Medical Group, Dalian, China; ^4^ Institute for Stem Cell and Regeneration, Chinese Academy of Sciences, Beijing, China; ^5^ CAS Center for Excellence in Brain Science and Intelligence Technology, Chinese Academy of Sciences, Shanghai, China

**Keywords:** placental model, organ-on-a-chip, trophoblast, perfused 3D culture, hiPSCs

## Abstract

The human placenta serves as a multifunctional organ to maintain the proper development of a fetus. However, our knowledge of the human placenta is limited due to the lack of appropriate experimental models. In this work, we created an *in vitro* placental trophoblast-like model via self-organization of human induced pluripotent stem cells (hiPSCs) in a perfused 3D culture macrofluidic device. This device allowed cell seeding, *in situ* trophoblast lineage differentiation, and formation of trophoblast-like tissues from hiPSCs in a biomimetic microenvironment. It incorporated extracellular matrix (ECM) and fluid flow in a single device. After trophoblast lineage differentiation, we were able to generate the 3D clusters with major cell types of the human placenta, including trophoblast progenitor cytotrophoblasts (CTBs), differentiated subtypes, syncytiotrophoblasts (STBs), and extravillous trophoblasts (EVTs) under long-term 3D culture (∼23 days). Moreover, the formed tissues exhibited enhanced expressions of CTB-, STB-, and EVT-related markers at the level of genes and proteins under a dynamic culture compared with static conditions. RNA-seq analysis revealed the higher expression of trophoblast-specific genes in 3D tissues, indicating the essential role of fluid flow to promote the trophoblast differentiation of hiPSCs. The established placental 3D model combined a bioengineering strategy with developmental principles, providing a promising platform for the study of placental biology in a biomimetic microenvironment in health and disease.

## 1 Introduction

The human placenta is an extremely essential and multifaceted organ that supports the development and maturity of the fetus (Adu-Gyamfi et al., 2021). Placental development dysfunctions may lead to a series of pregnancy complications, such as pre-eclampsia, fetal growth restriction (FGR), and preterm birth, which contribute to maternal and neonatal morbidity and mortality, etc. (Aplin et al., 2020). Placental trophoblast cells are specialized cells in the developing placenta (Okae et al., 2018). After implantation, the trophectoderm from the blastocyst rapidly proliferates and generates the trophoblast. *In vivo*, proliferative villous cytotrophoblast cells give rise to two main sub-populations, syncytiotrophoblasts (STBs) and extravillous trophoblasts (EVTs) (Xiao et al., 2020).

Presently, progresses of placental studies mostly come from animal models, cell line-based models, and stem cell-derived *in vitro* models (Shibata et al., 2020). Animal models such as mice have advanced the study of the molecular mechanisms of placental development and disease, but significant species difference exists between humans and animals (Hemberger et al., 2020). The monolayer cell culture models often use choriocarcinoma and immortalized cell lines which do not represent the near-physiological feature of the human placenta with complex cellular types and 3D architecture. Stem cells, especially human pluripotent stem cells (PSCs), have been used to generate trophoblast cells, thus providing unique insights into the studies of the human placenta *in vitro* (Xu et al., 2002; Amita et al., 2013; Horii et al., 2016)*.*

Organ-on-a-chip is a micro-physiological system that can mimic the key aspects of human organs *in vitro* and has been used for organ development studies (Homan et al., 2019), disease modeling (Qiu et al., 2018), and drug testing (Bhatia and Ingber, 2014; van Duinen et al., 2015; Ronaldson-Bouchard and Vunjak-Novakovic, 2018; Pemathilaka et al., 2019). Recently, several placenta-on-a-chip have been engineered to recapitulate the maternal–fetal interface (Blundell et al., 2016; Arumugasaamy et al., 2018; Yin et al., 2018; Zhu et al., 2018; Pemathilaka et al., 2019; Richardson et al., 2020; Boos et al., 2021). Most of these models use choriocarcinoma cells as cell sources (such as Bewo and JEG-3 cell lines) (Blundell et al., 2016; Arumugasaamy et al., 2018; Yin et al., 2018; Zhu et al., 2018). Despite the significant progress, the models that can represent the physiologically relevant features and microenvironment of the human placenta are still lacking. *In vivo*, the development of the placenta is a dynamic process and can be affected by multiple factors including mechanical cues and intrinsic or extrinsic biochemical signals (Burton et al., 2009; Miura et al., 2015).

In this work, we established a new approach to create a placental trophoblast-like 3D tissue model from the self-organization of hiPSCs in a perfused macrofluidic chip system. The device consisted of a central 3D culture chamber incorporated with fluid flow and extracellular matrix (ECM) that allowed for cell growth, *in situ* lineage differentiation, stem cell self-organization, and formation of placental trophoblast-like tissues in a biomimetic microenvironment. The formed tissues were characterized by the inclusion of major cellular components of the human placenta, including trophoblast progenitor cytotrophoblasts (CTBs) and differentiated subtypes, syncytiotrophoblasts (STBs), and extravillous trophoblasts (EVTs), and expressions of trophoblast-specific genes and proteins. The effects of the dynamic flow on the differentiation of hiPSCs into trophoblast lineage were assessed by immunofluorescent analysis, qRT-PCR, Western blot, and transcriptome analysis. The associated signaling pathways involved in regulating this differentiation process were discussed as well.

## 2 Materials and Methods

### 2.1 Fabrication of Macrofluidic Chips

The macrofluidic chip was fabricated from polymethyl methacrylate (PMMA) by a laser cutting process and a polydimethylsiloxane (PDMS) sheet via a customized cutting die technology. It consisted of four layers. The first and fourth layers were made of PMMA and used for chip housings. In cooperation with screws, the first and fourth layers were used for sealing the middle channel. A through-hole on the first layer is designed to make the medium inside more accessible and it was sealed with a rubber plug during perfusion. The middle two layers were the cell culturing chamber made of PDMS.

The stainless-steel tubes at the sidewalls of the second layer worked as the inlet and the outlet of the device. Their inner and outer diameters were 0.5 and 0.8 mm, respectively. The third layer was a glass slide bonded with a 0.5 mm thick PDMS sheet which was cut by the same customized cutting die technology. The third layer was pre-coated with 0.5 mm-thick Matrigel (BD, 356234). Matrigel would adhere to the sidewall and form a concave meniscus for the capillary effect. To decrease the waste of a large portion of the Matrigel, the second and third layers were designed to be separated rather than bonded. Then, the second layer was overlapped and chip housings were assembled to construct a leak-free fluid environment. Tubes were connected and a rubber plug was plugged in while perfusion was applied by using peristaltic pumps (Ditron Tech, BT100). Specifically, the width, length, thickness, cell culture area, and volume of the elliptic-shape chamber are 17.5 mm, 58.5 mm, 4 mm, 7.36 cm^2^, and 3 ml, respectively. The chip design and dimensions are given in Supplementary Figure S1.

### 2.2 Human Pluripotent Stem Cell Maintenance

The hiPSC cell line was kindly gifted by Ning Sun (Fudan University). According to the previous works of literature (Cui et al., 2022), the cell line was cultured in the mTeSR1 medium. When the cell reached 70–80% confluency, cells were passaged.

### 2.3 Induction and Differentiation of Human Pluripotent Stem Cells

Before seeding cells, ∼0.4 ml 100% Matrigel was added into the third layer of the chip which was assembled with the fourth layer of the chip previously. After Matrigel has solidified, the second, third, and fourth layers were subsequently assembled. To generate the trophoblast lineage, the dissociated hiPSCs (∼1 × 10^6^ cell) were resuspended with a 3 ml trophoblast induction medium (TIM) and poured into the Matrigel layer on the chip through the through-hole. The last step is that the first layer was integrated with the other parts to form a 3D culture device for organoid differentiation and formation. Based on the previous method for the establishment of trophoblast-like tissue (Cui et al., 2022), the TIM comprises DMEM/F12 (Gibco), 2% BSA (Meilunbio), 10 ng/ml BMP4 (Gibco), 1× ITS-X (Sigma), 100 ng/ml heparin (Sigma), 1× penicillin–streptomycin (Gibco), 1× MEM-NEAA (Gibco), and 1× L-GlutaMAX (Gibco). After 3 days of culture, the formed 3D clusters were cultured with a trophoblast organoid medium (TOM). The TOM comprises DMEM/F12 (Gibco), 1 × 1.25 mM N-acetyl-L-cysteine (Sigma), 2.5 μM prostaglandin E2 (PGE2) (Sigma), 2 μM Y-27632 (PeproTech), 1.5 μM CHIR99021 (Selleck), 80 ng/ml human R-spondin 1 (R&D systems), 50 ng/ml human EGF (PeproTech), N2 (Gibco), 1x B27 minus vitamin A (Gibco), 1× L-GlutaMAX (Gibco), 1× penicillin–streptomycin (Gibco), 500 nM A83-01 (Sigma), 50 ng/ml human HGF (PeproTech), and 100 ng/ml human FGF2 (PeproTech). The cells were cultured under the TIM medium from day 0 to day 3. From day 3 to day 23, the cells were cultured with the TOM medium. On day 5, the formed tissues under dynamic conditions were perfused at the rate of 1 ml/min. The static formed tissues were cultured under the same conditions as dynamic except without the inclusion of liquid flow. The medium was changed every 2–3 days. On day 23, the samples were collected for further analysis.

### 2.4 Primary Placental Tissues

Human primary placental tissues (6–8 weeks of gestation) were obtained from elective terminations. The use of these tissues was under ethical approval from the Ethics Committee of Dalian Municipal Women and Children’s Medical Center (No. 2019005). Guided by the principles in the Declaration of Helsinki of the World Medical Association, we obtained written informed consent from all participants. The obtained tissues were immediately fixed in 4% PFA for further immunofluorescence staining and HE staining.

### 2.5 Finite-Element Computational Simulations of Shear Stress and Particle Displacement Assay

The CAD model was established using SolidWorks and subsequently imported to COMSOL Multiphysics to perform the finite-element computational model. The velocity fields in the whole volume and multiple cross-sections were simulated. Specifically, the simulation was carried out by solving the incompressible Navier–Stokes equation. A no-slip condition was applied for all the surfaces except the inlet and outlet. The dynamic viscosity and density of the culture medium were set to be 0.69 mPa⋅s and 1 g/cm^2^, respectively. Furthermore, the shear stress exerted on the 3D tissues was simulated and presented using heatmaps and a line graph as given in Supplementary Figures S2A,B.

In addition, we performed a particle displacement assay using 10 μm microparticles within the system to cross-examine the exact flow rate with an experimental approach. Videos were recorded with a high-speed camera and images were further analyzed using imageJ. The resulting flow rate was 0.46 ± 0.03 mm/s in the central region of the chip (*n* = 3), which is close to the simulation result (0.44 mm/s). Since chip parts were produced using cutting dies and CNC machining, and the fabrication variances are within 0.5 mm. In addition, the chip is 58.5 mm long and 17.5 mm wide, which is much larger than fabrication variances. The resulting variances in the flow rate are negligible (Supplementary Video S1).

### 2.6 Flow Cytometry

According to the previous articles (Okae et al., 2018; Turco et al., 2018), the 3D tissues were collected in a centrifugal tube (Jet Biofil) and then removed from Matrigel using cell recovery solution (Corning 354253). Then the 3D tissues were dissociated with 0.25% trypsin into a single cell and passed through a 40 μm filter (Falcon 2340). For the flow cytometric analysis, the harvested cells were then incubated with a PE-conjugated anti-ITGA2 antibody for 15 min at room temperature. PE-conjugated mouse IgG2a was used as the isotype control. Flow cytometry was carried out using a SH800S Cell Sorter (Sony Biotechnology).

### 2.7 Immunofluorescence Staining

Before sample collection, the chip device was dismantled for obtaining the 3D placental trophoblast-like tissues conveniently. The collected 3D tissues were fixed in 4% paraformaldehyde (PFA) overnight. On day 2, the 3D trophoblast cultures were washed in PBS and dehydrated in 30% sucrose overnight. On day 3, the sucrose was removed and OCT (Sakura Finetek) was added to embed the 3D tissues at room temperature. After the bubbles were removed, they were snap-frozen and stored at −80°C. For immunohistochemistry, the 3D tissues were cut by using an ice cutter (Leica) of 10–15 μm. The Cryosection of the 3D tissues was washed with PBS to remove excess OCT and permeabilized with 0.2% Triton X-100 for 5–15 min. After that, the washed cryosection was blocked with 10% blocking serum for 1 h at room temperature. One hour later, the sections were incubated with primary antibodies diluted in antibody dilutions. On day 2, the primary antibodies were removed using PBS and the sections were incubated with secondary antibodies diluted in PBS for 1 h at room temperature. After incubation with secondary antibodies, the sections were performed in PBS. Finally, DAPI was performed to visualize the nucleus for 15 min at room temperature. After washing in PBS, the coverslips were mounted in sections with 50% glycerin and imaged using an Olympus FV1000 confocal laser-scanning microscope. The following primary antibodies were used for immunohistochemistry: P63 (mouse, 1:100, Abcam, ab735), GATA3 (mouse, 1:100, Santa Cruz Biotechnology, sc-268), HLA-G (mouse, 1:500, Santa Cruz Biotechnology, sc-21799), ENDOU (rabbit, 1:500, Sigma, HPA012388), KRT7 (mouse, 1:100, Thermo Fisher Scientific, MA5-11986), hCG-β (rabbit, 1:300, Abcam, ab53087), ZO-1 (rabbit, 1:100, Abcam, ab221547), and GLUT-4 (rabbit, 1:100, Abcam, ab654). The secondary antibodies were Alexa Fluor 488- and 594 conjugated anti-donkey (CST, 1:500).

### 2.8 Western Blot Analysis

The 3D tissues were collected and lysed immediately on ice using the RIPA lysis buffer system (Beyotime, P0013K). The lysates were diluted in a 2× loading buffer and warmed at 95°C. The lysates were separated on gels and transferred to a PVDF membrane (GE Amersham) which was then incubated at 4°C overnight with the primary antibodies: ENDOU (rabbit, 1:500, Sigma, HPA012388) and hCG-β (rabbit, 1:300, Abcam, ab53087). Subsequently, the membranes were incubated with HRP-conjugated secondary antibodies for 1 h at room temperature. Protein bands were visualized with a Prime Western Blotting Detection Reagent (GE life) in ChemiDoc XRS+ System (Bio-Rad).

### 2.9 qRT-PCR

The RNAiso Plus (Takara) was used to extract mRNA which was then dissolved in RNAase-free water. The mRNA was converted to cDNA using the reagent PrimeScript RT Master Mix (Takara). qRT-PCR was performed on PikoReal 96 using Ex Taq DNA polymerase (Takara) under the following reaction conditions: 40 cycles of denaturation at 94°C for 30 s, annealing at 58°C for 45 s, and extension at 72°C for 30 s. The primers used in this work are given in Supplementary Table S1.

### 2.10 RNA Sequencing

According to pervious articles (Cui et al., 2020; Zhao et al., 2020), the mRNA of 3D tissues was extracted and purified with Oligo (dT)-conjugated magnetic beads. The purified mRNA was fragmented into small pieces using fragment buffer at an appropriate temperature. Reverse transcription using a random hexamer primer was performed to generate the first-strand cDNA and the second-strand cDNA. Subsequently, A-Tailing Mix and RNA Index Adapters were added and incubated to perform end repair. The obtained cDNA fragments were amplified by PCR and the generated products were purified using Ampure XP Beads, which were then dissolved in EB solution. The products were assessed using the Agilent Technologies 2100 bioanalyzer for quality control (QC). To obtain the library, the previously obtained double-stranded PCR products were denatured and circularized using the splint oligo sequence. The final library was generated through the formatting of the single strand circle DNA (ssCir DNA). Subsequently, a DNA nanoball (DNB), with more than 300 copies of one molecule, was generated by amplifying the final library with phi29. Finally, the produced DNBs were loaded into the patterned nanoarray of the BGIseq500 platform (BGI-Shenzhen, China) to generate single-end 50 base reads. The DEGs (differentially expressed genes) were defined by the criteria that the *p*-value threshold after correction for multiple testing (adjusted *p*-value) was set to 0.01 and the threshold of fold change in mRNA expression was 4. Gene Ontology (GO) and Kyoto Encyclopedia of Genes and Genomes (KEGG) enrichment analyses of DEGs were both performed using the Dr. Tom system from BGI.

### 2.11 Statistical Analysis

Data were expressed as the means ± SEM and analyzed by performing Student’s *t*-test. Significance levels were denoted as follows: *p < 0.05, **p < 0.01, and ***p < 0.001. Sample sizes were indicated in the figure legends. The expression level of each gene was compared with that of the house-keeping gene GAPDH using the comparative ΔΔCT method. Image-Pro Plus 6.0. was used to quantify the immunofluorescence intensity of the images. In addition, the data were handled with Excel, AI, and GraphPad Prism.

### 2.12 Data Availability

Data files of RNA-sequencing in this study are accessible at SRA with the accession number PRJNA713853. A link to BioProject’s metadata is ready to be shared with reviewers listed in the following sections (https://dataview.ncbi.nlm.nih.gov/object/PRJNA713853?reviewer=2cvm6ss9324tacfidrb2q353l1). All other data that support the findings of this study are available from the corresponding authors.

## 3 Results and Discussion

### 3.1 Design and Operation of the Macrofluidic Chip System for Culturing 3D Tissue

*In vivo*, the placenta is in a complex cellular and mechanical microenvironment which can affect cellular phenotypes and functions. The placental cells interact with the surrounding extracellular matrix (ECM) including collagens, laminins, and proteoglycans, which play a critical role in regulating a normal and pathological pregnancy (O'Connor et al., 2020). In addition, mechanical cues such as fluid shear stress are essential for the development of the placenta, especially during the onset of maternal blood flow into the intervillous space toward the end of the first trimester (Morley et al., 2019).

**FIGURE 1:**
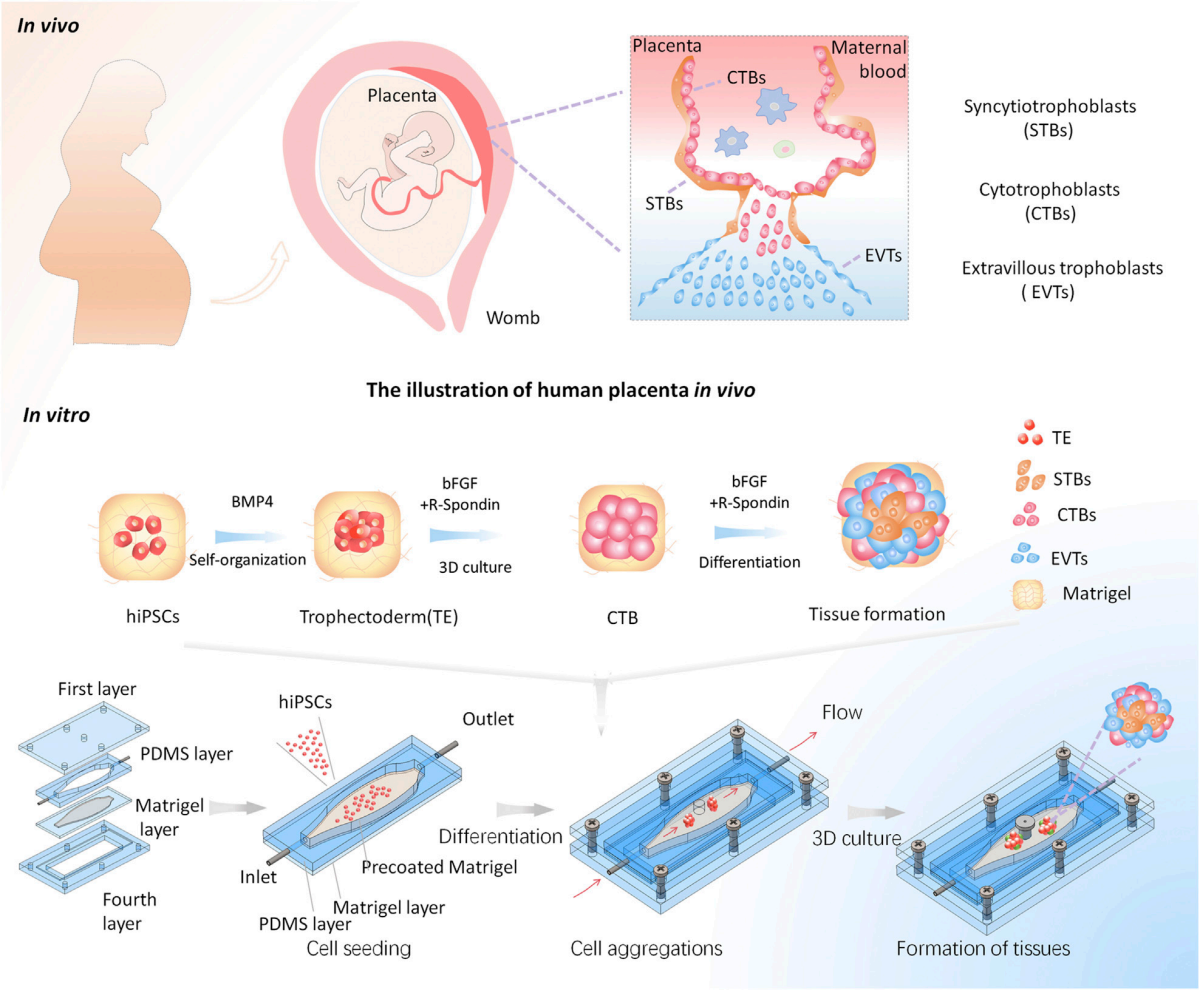
Schematic illustrations of the generation of the placental trophoblast-like tissue model from hiPSCs in a macrofluidic chip. The human placental trophoblasts *in vivo* comprise villous CTBs, an outer layer of multinucleated STBs, and EVTs. The differentiation process of hiPSCs toward trophoblast lineage on the chip mainly includes three stages: hiPSC seeding, cell aggregations, and formation of trophoblast tissues under a dynamic 3D culture.

To mimic the *in vivo*-like placental tissue microenvironment, we established a macrofluidic chip based on the previous work (Homan et al., 2019), which can enable the formation of trophoblast tissues in a biomimetic 3D microenvironment including dynamic flow and 3D ECM. It comprised four layers (Figure 1). The first and fourth layers were chip housings used for sealing the middle channels. In particular, a through-hole on the first layer of the macrofluidic chip was designed for cell seeding and sample collection for subsequent analysis. The second layer was a 4 mm thick PDMS sheet to serve as the perfused channel wall. The central culture chamber was designed with a tapered and elliptic shape, thus enabling the growth of cells and tissues under homogeneous fluid velocity. The third layer was a 0.5 mm thick PDMS sheet bonded with a glass slide, which enables the pre-coating of a thin Matrigel before the assembly of the device. It could reduce the capillary effect to maintain the even distribution of Matrigel. The second and third layers were assembled to form the elliptic-shaped chamber for cell culture. This device enables the growth of tissue in 3D Matrigel within a leak-free fluid environment, which could be directly exposed to a homogeneous flow condition. Moreover, the system allows for cell aggregation, *in situ* differentiation, and self-organization of hiPSCs into trophoblast-like tissues under a perfused 3D culture condition on a single device. It will facilitate the *in situ* tracking and real-time imaging of the growth of 3D tissues and subsequent analysis.

The human placenta *in vivo* is surrounded by dynamic flows of maternal blood. To better recapitulate the *in vivo*-like flow microenvironment, medium perfusion was applied to introduce physiologically relevant shear stress on the cell culture region under dynamic conditions. It has been previously reported that the placenta can produce more placental growth factors in a biofluidic environment with blood flow (Miura et al., 2015). Moreover, the placental cells can be fused within the physiological range of shear stress: 0.001–2.3 dyn/cm^2^ (Sanz et al., 2019). To achieve physiologically relevant shear stress, we performed a perfusion culture at the flow rate of 1 ml/min. A finite element analysis showed that the resulting shear stress is 0.0028 dyn/cm^2^, which fell within the physiological range of shear stress as present *in vivo*. It is noted that the fluid velocity distribution was relatively uniform in the cell culturing area under this condition (Supplementary Figure S2A). Furthermore, the homogenous distribution of shear stress was obtained in the elliptic-shaped cell culturing area (Supplementary Figure S2B). This condition might facilitate better recapitulation of a dynamic environment condition which is similar to what is experienced *in vivo.*

### 3.2 Characterization of Trophoblast Differentiation From hiPSCs on Chip Under Static Conditions

**FIGURE 2:**
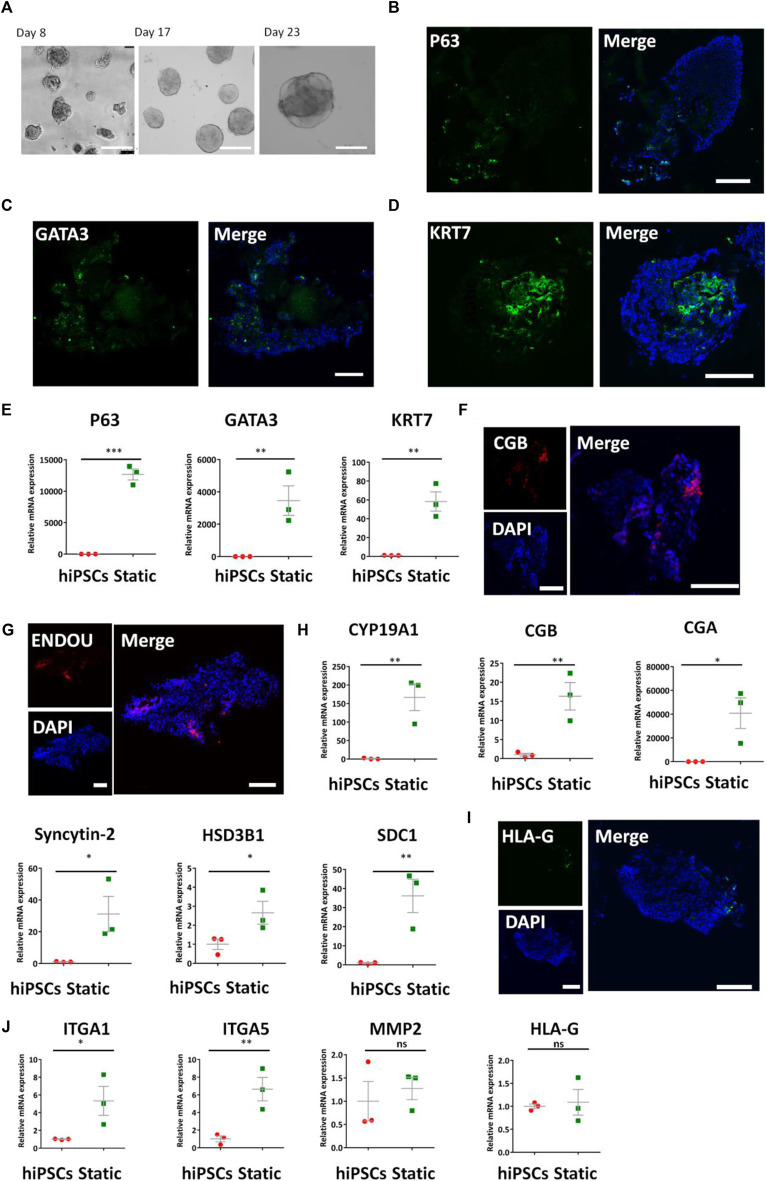
Expression of CTB-, STB-, and EVT-specific markers in the formed 3D tissues under static conditions. **(A)** Bright-field images of the growth of 3D tissue on different days (8, 17, and 23) after exposure to a medium with a cocktail of defined factors. Scale bars: 200 μm. **(B–D)** Immunofluorescence analysis of CTB markers **(B)** P63, **(C)** GATA3, and **(D)** KRT7 in 3D tissues under static conditions. **(E)** Transcript levels of p63, GATA3, and KRT7 were analyzed by qRT-PCR. Data were mean ± SEM (*n* = 3). **(F,G)** Representative immunofluorescent images showing the STB-associated markers ENDOU and CGB under static conditions. **(H)** qRT-PCR analyses were performed to detect the STB-related markers (listed in figure) under static conditions. Data were mean ± SEM (*n* = 3). **(I)** Immunofluorescent images indicated HLA-G expression static conditions. **(J)** EVT-related markers (listed previously) were detected by qRT-PCR. GAPDH was used as the reference gene. The data were analyzed using Student’s *t-*test (*p < 0.05; **p < 0.01; and ***p < 0.001). Scale bars: 100 μm unless otherwise stated.

To obtain the optimized condition for constructing the placental trophoblast tissue model, we initially induced the trophoblast differentiation of hiPSCs under static 3D culture conditions on a chip. As BMP4 is reported to facilitate the differentiation of trophoblast from hiPSCs (Xu et al., 2002; Amita et al., 2013; Li et al., 2013; Horii et al., 2016), we used the differentiation protocols in a defined medium with BMP4 in Matrigel under static conditions. Initially, we seeded hiPSCs on the adherent Matrigel layer on the bottom of the chip and allowed the self-assembly of hiPSCs with the medium supplemented with BMP4 for 3 days. Then, we treated the developing 3D tissues from day 3 to day 23 with a defined cocktail of factors including R-spondin 1, human HGF, and human EGF, etc., which has been reported to promote the differentiation of trophoblast-like lineage (Cui et al., 2022). As shown in Figure 2A, the bright-field images revealed the presence of cell clusters from hiPSCs in 3D conditions at different times.

To identify the cell types within the 3D cultures, we performed a series of analyses as identified by qRT-PCR, immunofluorescent staining, and Western blot. Cytotrophoblasts (CTBs) represent an undifferentiated and proliferative population (Okae et al., 2018). They express GATA3, KRT7, and P63 (Lee et al., 2007). Immunofluorescent staining indicated the presence of positive P63, GATA3, and KRT7 in the 3D tissues (Figures 2B–D). P63, KRT7, and GATA3 have been found in human primary tissues (6–8 weeks) (Supplementary Figures S3A–C). In addition, we recognized the upregulated expression of CTB-related genes P63, GATA3, and KRT7 in the tissue by qRT-PCR compared with hiPSCs (Figure 2E).

Syncytiotrophoblasts (STBs) are a multinucleated epithelium of the villi and participate in nutrient exchange and hormone production (Peng et al., 2004; Kudaka et al., 2008; Fournier et al., 2015; Suryawanshi et al., 2018). Immunofluorescence staining results revealed the expression of STB-specific markers ENDOU and CGB in the 3D tissues under static conditions (Figures 2F,G). The human primary tissues exhibited the existence of STB-specific markers ENDOU and CGB (Supplementary Figures S3D,E). The qRT-PCR analysis further analyzed the expression of STB-related genes CGA, CGB, Syncytin-2, HSD3B1, and CYP19A1, etc., in trophoblast-like tissues compared with hiPSCs (Figure 2H).

It is known that the sublineage markers of EVTs contain HLA-G. We next examined the differentiation of EVTs from hiPSCs under this device without perfusion. In line with the presence of HLA-G + cells in human primary tissues (6–8 weeks) (Supplementary Figure S3F), the 3D tissues exhibited few HLA-G expressions assessed by immunofluorescence analysis (Figure 2I). Moreover, we detected expressions of EVT-specific genes HLA-G, MMP2, ITGA1, and ITGA5 in 3D tissues by qRT-PCR (Figure 2J). It validated the trend of upregulated expression of these genes compared with the hiPSCs. Altogether, it appears that the generated 3D tissues under static conditions include typical trophoblast multiple cellular types, including cytotrophoblasts (CTBs), differentiated subtypes, syncytiotrophoblasts (STBs), and extravillous trophoblasts (EVTs).

### 3.3 Enhanced Trophoblast Differentiation of hiPSCs in the Perfusable 3D Culture Chip

**FIGURE 3:**
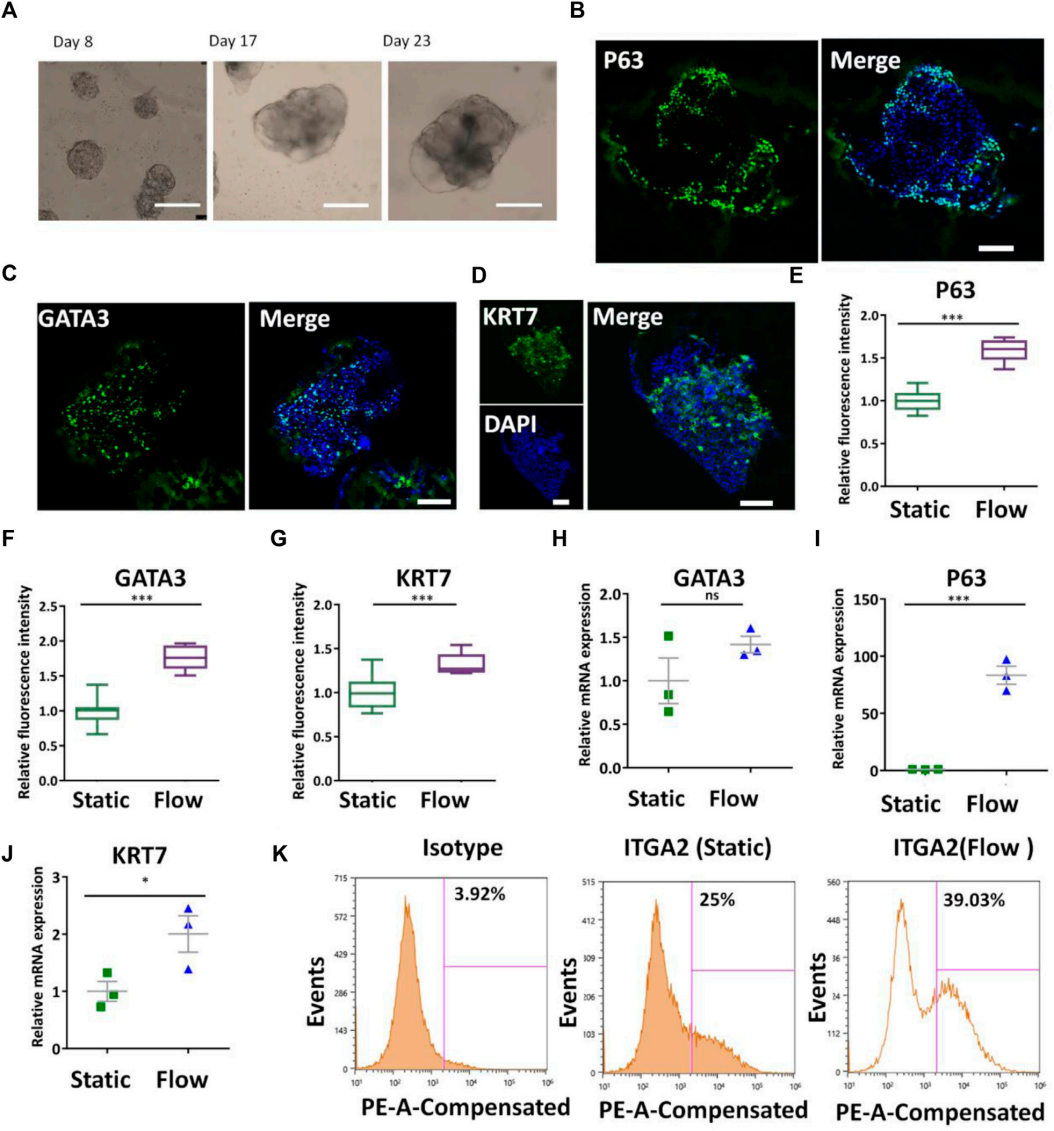
Differentiation of CTBs in the formed tissues under dynamic conditions. **(A)** Bright-field images of the growth of 3D tissue on different days (8, 17, and 23) after exposure to a medium with a cocktail of defined factors at the perfusion of 1 ml/min. Scale bars, 200 μm. **(B–D)** Immunofluorescence image of the analysis of CTB markers P63, GATA3, and KRT7 under dynamic conditions. Scale bars, 100 μm. **(E–G)** P63^+^, GATA3^+^, and KRT7^+^ cells were quantified in 3D tissues under static and dynamic conditions. The data are indicated as mean ± SEM (*n* > 3). **(H–J)** Expression levels of P63, GATA3, and KRT7 genes in 3D tissues under different conditions were probed by qRT-PCR. Data were mean ± SEM (*n* = 3). GAPDH was used as the reference gene. **(K)** ITGA2^+^ cell population in 3D tissues under different culture conditions was quantified by flow cytometry analysis. The data were analyzed using Student’s *t-*test (*p < 0.05; **p < 0.01; and ***p < 0.001).

The placenta is a mechanosensitive organ and fluid shear stress is essential for the proper development of the placenta. To investigate the effects of fluidic flow on the trophoblast differentiation of hiPSCs, the cells were initially seeded in the cell culture chamber and cultured for 4 days to enable the adherence of the cells on the Matrigel layer. Then they were perfused with fluidic flow (1 ml/min) to allow shear stress stimulation and nutrient supply on the growing 3D tissues. The bright-field images showed the different morphologies and sizes of 3D tissues on days 8, 17, and 23 under dynamic conditions (Figure 3A) It seemed that the size of 3D tissues under dynamic conditions was bigger than those under static conditions (quantitative data not shown). HE staining showed the morphology of primary tissues obtained from elective terminations and the trophoblast-like tissues under dynamic or static conditions (Supplementary Figure S4). It showed a significant difference in 3D tissues under static or dynamic conditions with the primary placental tissue. In particular, the 3D tissues under dynamic conditions were more similar to the primary placental tissue compared with those under static conditions. Then, trophoblast-specific genes and proteins in 3D tissues under different conditions were identified by qRT-PCR and immunofluorescence analysis. First, the CTB-specific proteins were analyzed by detecting the expression of P63, GATA3, and KRT7 via immunofluorescence staining (Figures 3B–D). Quantitative analysis proved the increased expression of P63, GATA3, and KRT7 in the formed 3D tissues under dynamic conditions (Figures 3E–G). In addition, it exhibited the trend of increased expression of CTB-specific genes P63, GATA3, and KRT7 under dynamic conditions identified by qRT-PCR (Figures 3H–J). *In vivo*, the proliferative trophoblast expressed ITGA2 marking cells at the base of the cytotrophoblast cell columns (Lee et al., 2018). We conducted a flow cytometry assay to quantify the proportion of ITGA2 in the 3D tissues under different conditions. It confirmed a higher percentage of ITGA2^+^ cells in 3D cultures under dynamic conditions (Figure 3K). All the aforementioned results revealed the role of fluidic flow to promote differentiation of CTBs in 3D tissues from hiPSCs potentially.

**FIGURE 4:**
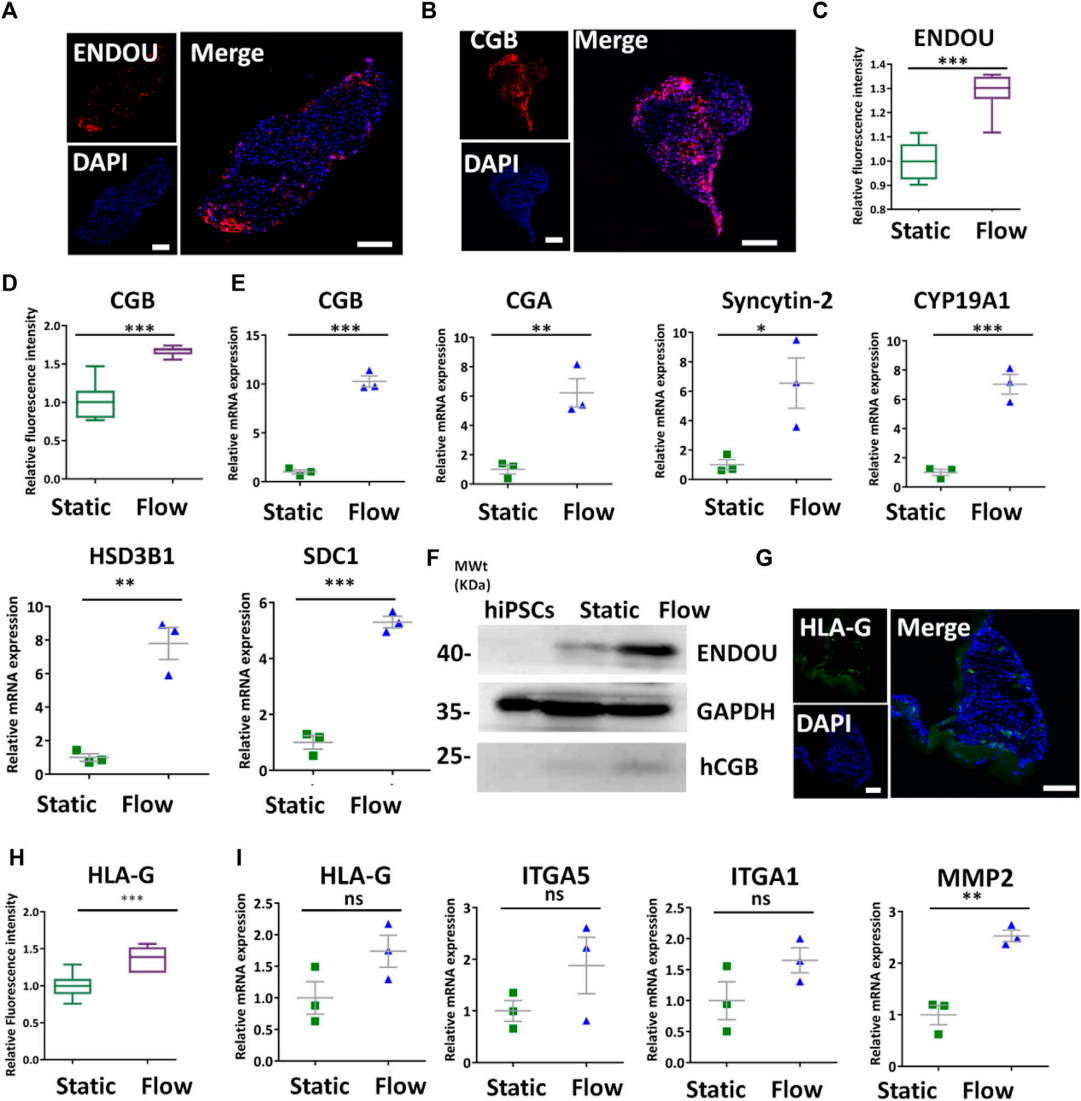
Characterization of STBs and EVTs’ differentiation in 3D tissues under dynamic conditions. **(A,B)** Representative immunofluorescent images showing the STB-associated markers ENDOU and CGB under dynamic conditions at the rate of 1 ml/min. Scale bars, 100 μm. **(C,D)** ENDOU^+^ and CGB^+^ cells were quantified in 3D tissues under dynamic and static conditions. The data are indicated as mean ± SEM (*n* > 3). **(E)** qRT-PCR analyses were performed to detect the STB-related markers (listed in figure) under static and dynamic conditions. **(F)** Western blot revealed more STB marker expressions under dynamic conditions compared with those found in hiPSCs and static conditions. **(G)** Immunofluorescent images indicated HLA-G expression under dynamic conditions. Scale bar = 100 μm. **(H)** HLA-G^+^ was quantified in 3D cultures under different conditions. The data are indicated as mean ± SEM (*n* > 3). **(I)** EVT-related markers (listed previously) were detected by qRT-PCR. GAPDH was used as the reference gene. Data were mean ± SEM (*n* = 3). The data were analyzed using Student’s *t*-test (*p < 0.05, **p < 0.01, and ***p < 0.001). Scale bars, 100 μm.

The CTBs can give rise to trophoblast subpopulations of STBs and EVTs. Therefore, we compare the differentiation of STBs under different conditions. It appeared that ENDOU^+^ and CGB^+^ cells in formed tissues under dynamic conditions assessed by immunofluorescence staining (Figures 4A,B). In addition, it revealed the increased expression of CGB and ENDOU under dynamic conditions compared with static conditions by quantitative analysis (Figures 4C,D). The qRT-PCR analysis revealed the enhanced expression of STB-related genes SDC1, CGA, CGB, HSD3B1, Syncytin-2, and CYP19A1 in the 3D tissues under flow conditions (Figure 4E). Moreover, it indicated the increased expression of ENDOU and CGB in 3D tissues under dynamic conditions detected by Western blot (Figure 4F), reflecting the favorable differentiations of STBs under the dynamic condition. Because of the critical role of STBs in nutrient transport and metabolism, we detected the GLUT transporter, GLUT4, to probe the glucose uptake under dynamic conditions. It revealed increased glucose uptake under dynamic conditions (Supplementary Figure S5). In addition to STBs, we examined the differentiation of EVTs under fluidic conditions. It demonstrated that 3D tissues exhibited higher HLA-G^+^ under flow conditions detected by immunofluorescence analysis (Figure 4G). Accordingly, it revealed the increased expression of HLA-G^+^ cells in 3D clusters under dynamic conditions by fluorescence intensity analysis (Figure 4H). In addition, we observed the tendency of the increased expression of EVT-specific genes in 3D tissues under dynamic flow conditions by qRT-PCR (Figure 4I), thus implying that the flow promoted the differentiation of EVTs in 3D tissues.

### 3.4 Transcriptome Analysis of the Produced Trophoblast-Like Tissue

**FIGURE 5:**
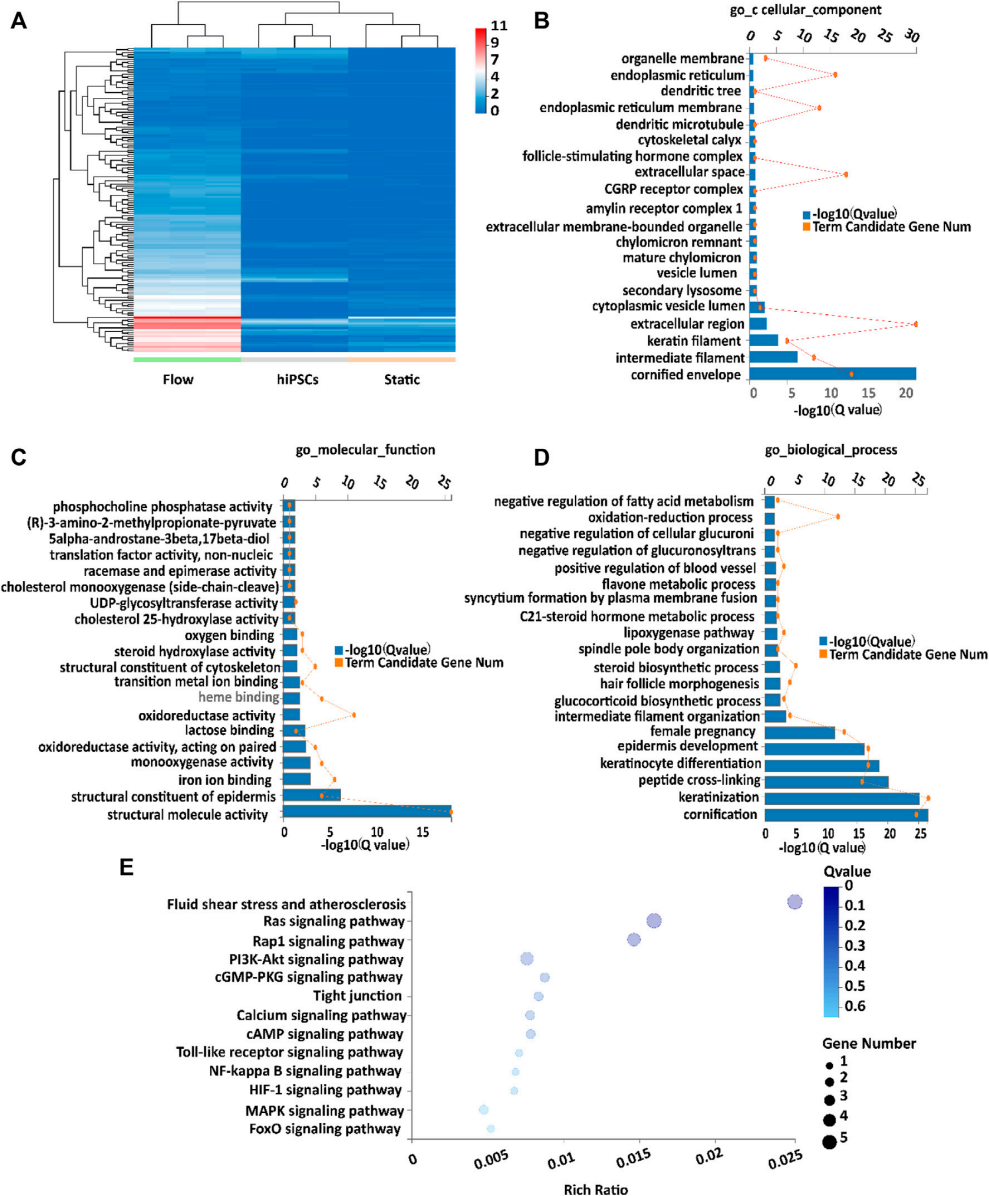
Transcriptome analysis of placental trophoblast-like tissues under static and dynamic conditions. **(A)** Hierarchical clustering heatmap of upregulated DEGs in the flow group compared with the static group, the undifferentiated hiPSC group. **(B–D)** GO enrichment of upregulated DEGs according to the **(B)** cellular component, **(C)** molecular function, and **(D)** biological process. **(E)** KEGG analysis of upregulated DEGs in 3D tissues under dynamic conditions compared with static conditions.

To gain more insights into the mechanisms of differentiation of placental trophoblast-like tissues, RNA-seq analysis was performed in the 3D tissues under flow and static conditions. The aforementioned results revealed the increased trend of expression of CTB-, STB-, and EVT-specific markers under flow conditions (Figures 3, 4). RNA-seq confirmed that dynamic flow enhanced the expression of CTB-, STB-, and EVT-specific genes compared with static conditions (Supplementary Figure S6). In this study, the DEGs (differentially expressed genes) in the dynamic flow group were defined by the criteria that the *p*-value threshold after correction for multiple testing (adjusted *p*-value) was set to 0.01 and the threshold of fold change in mRNA expression was 4. Based on the aforementioned criteria, the expression level of DEGs under different groups was demonstrated in hierarchical clustering (Figure 5A). The GO (GO enrichment) analysis of upregulated DEGs in dynamic flow conditions was involved in cellular components, such as the cellular membrane, endoplasmic reticulum, keratin filament, molecular function, including binding and phosphatase, and biological processes such as metabolism, keratinization, and keratinocyte differentiation (Figures 5B–D). It is in agreement with the previous study which indicated that shear stress is important for cellular metabolism and differentiation (Brugger et al., 2020).

**FIGURE 6:**
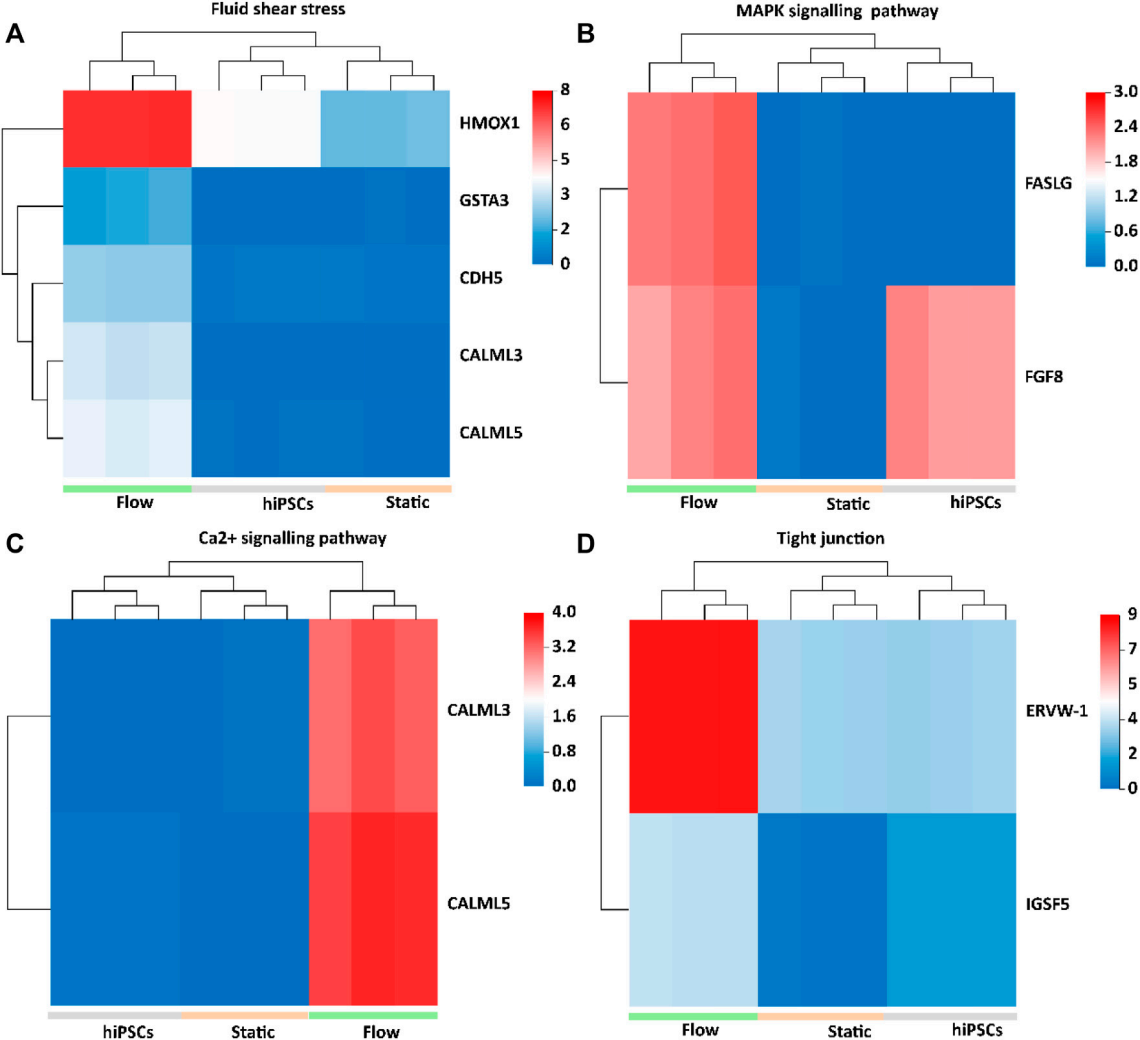
Heatmap of upregulated DEGs in 3D tissues under the flow condition, which were implicated in fluid shear stress, MAPK signaling pathways, Ca2^+^ signaling pathway, and tight junction. **(A)** HMOX1, GSTA3, CDH5, CALML3, and CALML5 upregulated in 3D tissues were enriched in fluid shear stress pathways. **(B)** FASLG and FGF8 upregulated in 3D tissues were enriched in the MAPK signaling pathway. **(C)** CALML3 and CALML5 were enriched in the Ca^2+^ signaling pathway. **(D)** ERVW-1, crucial for STB infusion, and IGSF5, a tight adhesion protein, were enriched in the tight junction.

**FIGURE 7:**
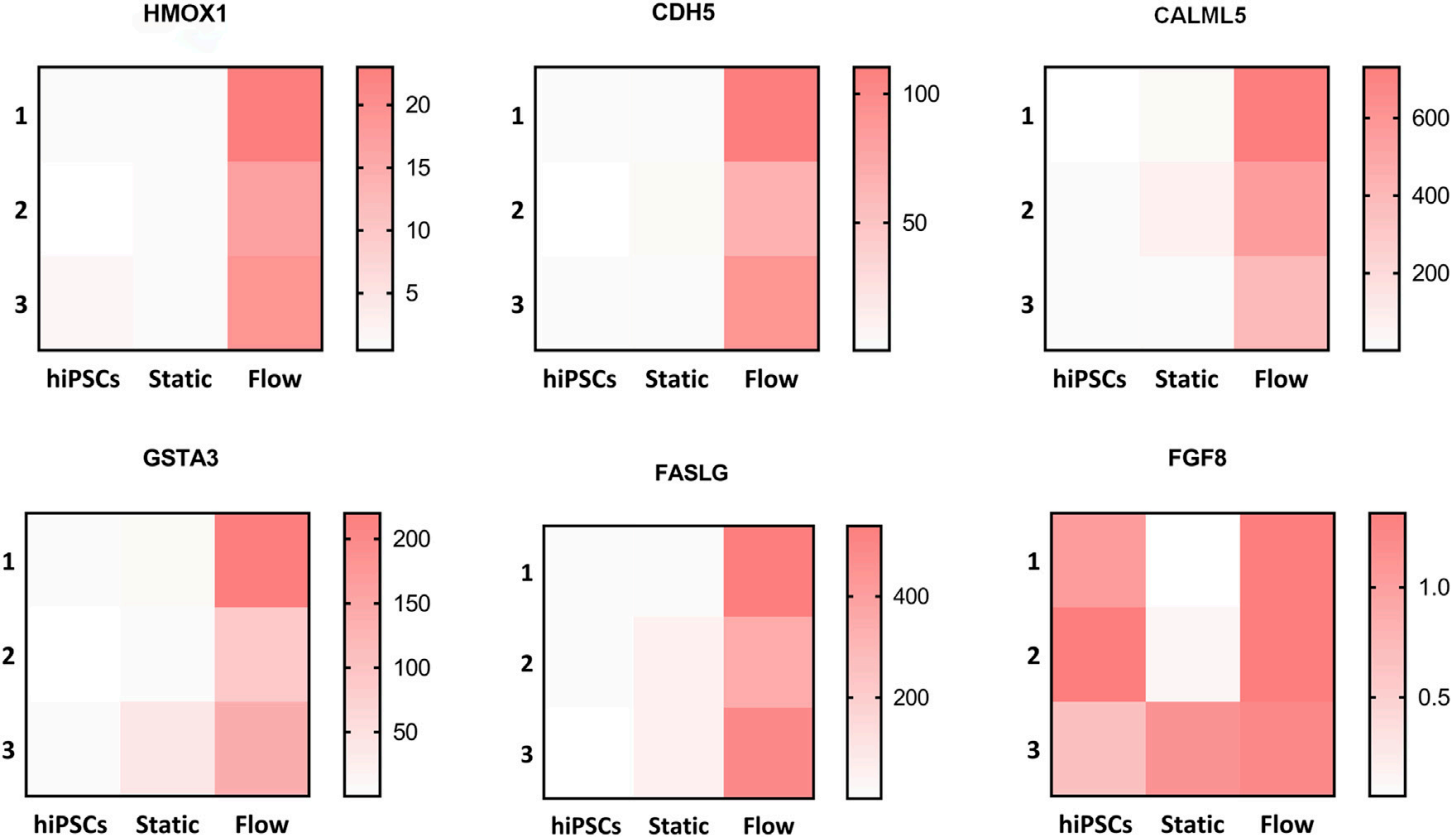
Altered genes involved in different pathways were validated via qRT-PCR. Validation of the relative mRNA expression of HMOX1, CDH5, CALML5, GSTA3, FASLG, and FGF8 involved in pathways such as the fluidic stress pathway, MAPK signaling pathway, Ca^2+^ signaling pathway, and tight junction. The altered genes were displayed in the form of a heatmap using GraphPad Prism.

The Kyoto Encyclopedia of Genes and Genomes (KEGG) analysis confirmed that the upregulated DEGs were involved in fluid shear stress, the MAPK signaling pathway, tight junction, and Ca^2+^ signaling pathway (Figure 5E). We next analyzed the specific genes involved in fluid shear stress (HMOX1, GSTA3, CDH5, CALML3, and CALML5), the MAPK signaling pathway (FASLG and FGF8), tight junction (ERVW-1 and IGSF5), and calcium signaling pathway (CALML3 and CALML5) using a heatmap (Figures 6A–D). It appeared that trophoblast differentiation might be regulated by the activated Ca^2+^ signaling pathway in dynamic flow conditions. This was in agreement with the previous study, in which the Ca^2+^ signaling pathway could regulate the mechanical responses of cells to continuous shear stress (Ugurel et al., 2021). In addition, some of the genes (HMOX1, CDH5, CALML5, GSTA3, FASLG, and FGF8) enriched in the pathways were validated by qRT-PCR and demonstrated in the format of a heatmap (Figure 7). These results reflected a similar trend to the expression profiling shown in the heatmap by RNA-seq analysis (Figure 6).

CHD5, known as VE-cadherin, is expressed in various cell types of trophoblasts and spiral artery endothelial cells (Adu-Gyamfi et al., 2021). ERVW-1 is essential for the fusion of STBs (Hlw et al., 2021). Compared with other groups, the increased expression of ERVW-1 and CDH5 was demonstrated in the formed tissues under dynamic conditions. In addition, we detected the tight junction protein ZO-1 in 3D cultures under different conditions. The 3D tissues under dynamic conditions exhibited better morphology in ZO-1 compared with those under static conditions (Supplementary Figure S7). It was assumed that the increased expression of ERVW-1, CDH5, and ZO-1 can activate the pathway of cell tight junction and shear stress pathways to promote the differentiation of trophoblast tissues under dynamic conditions in this study. In addition, it was proposed that the dynamic flow might facilitate the formation of matured placental tissue by enhancing the expressions of vascular tight junction proteins in trophoblasts in this study.

In this study, we demonstrated that dynamic flow, namely, shear stress, facilitated the differentiation of hiPSCs into trophoblast lineages. Different from our previous study which demonstrates that hiPSCs embedded in Matrigel can be differentiated toward trophoblast lineage under a defined medium condition (Cui et al., 2022), this work mainly focuses on the establishment of a biomimetic placental model by incorporating dynamic flow and 3D matrix.

A previous work indicated that shear stress can modulate trophoblast microvilli formation by characterizations of STBs and EVTs (Miura et al., 2015). However, we did not observe the microvilli formation for characterizations of STBs and EVTs in this study. In our subsequent work, we will be devoted to exploring the exact mechanism of shear stress modulating the trophoblast villi formation such as the analysis of TRP mechanosensors. In terms of the effects of shear stress on the invasion of EVTs, we aim to probe the effect of dynamic flow on the invasive properties of EVTs thoroughly in future.

In addition, the maturity and function of this trophoblast-like tissue remained to be improved compared with primary placental tissue. Structurally, the placenta is a complex and heterogeneous organ which comprises functional trophoblast cells and stromal cells. The stromal cells consist of macrophages, mesenchymal stromal cells, fibroblasts, and fetal endothelial cells (Li et al., 2020). These cells interact with each other to support the maturation and function of the human placenta. Furthermore, more complex and flexible engineered elements such as co-culture compartments would be incorporated into the established device to facilitate the maturity and function of trophoblast-like tissue. Furthermore, a more detailed examination of the culture conditions is required to explore whether it promotes the subsequent differentiation from CTBs to STBs and EVTs.

## 4 Conclusion

In this work, we established a new approach to construct a placental trophoblast 3D model from hiPSCs in a perfused macrofluidic chip. The produced 3D tissues comprised major cell types of trophoblasts, including CTBs, STBs, and EVTs, thus recapitulating the near-physiological feature of the human placenta *in vivo*. Furthermore, the 3D tissues exhibited increased expression of placenta-specific CTB-, STB-, and EVT- markers at the gene and protein levels under dynamic conditions, reflecting the essential role of fluid flow in promoting the trophoblast lineage differentiation from hiPSCs. An RNA-seq analysis revealed the trophoblast differentiation of the 3D tissue from hiPSCs under a 3D dynamic culture, which was associated with shear stress, Ca^2+^ pathways, and tight junction signaling pathways.

The novelty of this study is to produce a tissue model which can represent the multi-cellular components of placental tissue from hiPSCs within a biomimetic 3D environment. In addition, the established approach can integrate cell seeding, *in situ* differentiation, and self-organization of hiPSCs into trophoblast-like tissues under a perfused 3D culture on a single device, thus facilitating the *in situ* tracking and real-time imaging of the growth of 3D tissues and subsequent analysis.

To our knowledge, this is the first attempt to generate a hiPSC-derived 3D placental-like tissue model on a perfused chip by combining the engineering strategy with developmental principles. This established model can not only facilitate understanding the role of mechanical cues in the formation of placental trophoblast tissues but also provides a promising tool for investigating placental biology in a biomimetic microenvironment in normal tissues and diseases.

## Data Availability

The datasets presented in this study can be found in online repositories. The names of the repository/repositories and accession number(s) can be found at: https://www.ncbi.nlm.nih.gov/, PRJNA713853.

## Supplementary Material

Supplementary Figure S1 Chip design of the macrofluidic chip. The unit of numbers in this image is mm.

Supplementary Figure S2 Computational simulations of shear stress within the 3D culture region of the macrofluidic chip. **(A,B)** Characterization of flow features within the tapered and elliptic-shaped culture chamber of the device. The velocity fields in the whole volume and multiple cross-sections were simulated **(A)**. Shear stress exerted on the cells was simulated and presented in the form of heatmaps and a line graph. The flow rate is 1 ml/min. Shear stress within the culture was calculated to be 2.8 × 10^−4^ Pa (0.0028 dye/cm^2^) **(B)**.

Supplementary Figure S3 Characterization of trophoblast subtypes in human primary placental tissues (6–8 weeks). **(A–C)** Representative immunofluorescence pictures of **(A)** P63, **(B)** KRT7, and **(C)** GATA3 in the primary placental tissue (6–8 weeks). Scale bar=100 μm. **(D,E)** STB-related markers ENDOU and CGB were detected by immunofluorescence stain in the primary placental tissue (6–8 weeks). **(F)** Immunofluorescence analysis was performed to detect the expression of HLA-G in primary tissue (6–8 weeks).

Supplementary Figure S4 HE staining of primary placental (6–8 weeks), and trophoblast-like tissues under flow and static conditions. Scale bars, 200 μm.

Supplementary Figure S5 Immunofluorescence images of GLUT4 in trophoblast-like tissues under flow and static conditions. Scale bars, 20 μm.

Supplementary Figure S6 Heatmap of altered CTB-, STB-, and EVT-related markers in placental trophoblast-like tissues derived from hiPSCs under static and dynamic conditions from RNA-seq analysis. **(A)** Expression of CTB-specific genes GATA3, KRT7, TEAD4, ELF5, and TFAP2C was demonstrated in a heatmap in RNA-seq. **(B)** STB-related genes SDC1, CGA, CYP19A1, ERVW-1, LGAALS13, SLC1A1, PSG1, DEPDC18, ADHFE1, INHA, MUC15, GCM1, INSL4, and HSD31B were displayed in a heatmap calculated by FPKM. **(C)** EVT-related markers ITGA5, ITGA1, MMP11, SPON2, and PAPPA1 were demonstrated in a heatmap from RNA-seq analysis.

Supplementary Figure S7 Immunofluorescence images of ZO-1 in trophoblast-like tissues under flow and static conditions. Scale bars, 20 μm.
