# Supplementary figures and images for: Fluidic Flow Enhances the Differentiation of Placental Trophoblast-Like 3D Tissue from hiPSCs in a Perfused Macrofluidic Device
Source: Front Bioeng Biotechnol. 2022 Jun 30;10:907104. doi: 10.3389/fbioe.2022.907104 (PMC9280037; doi:10.3389/fbioe.2022.907104)

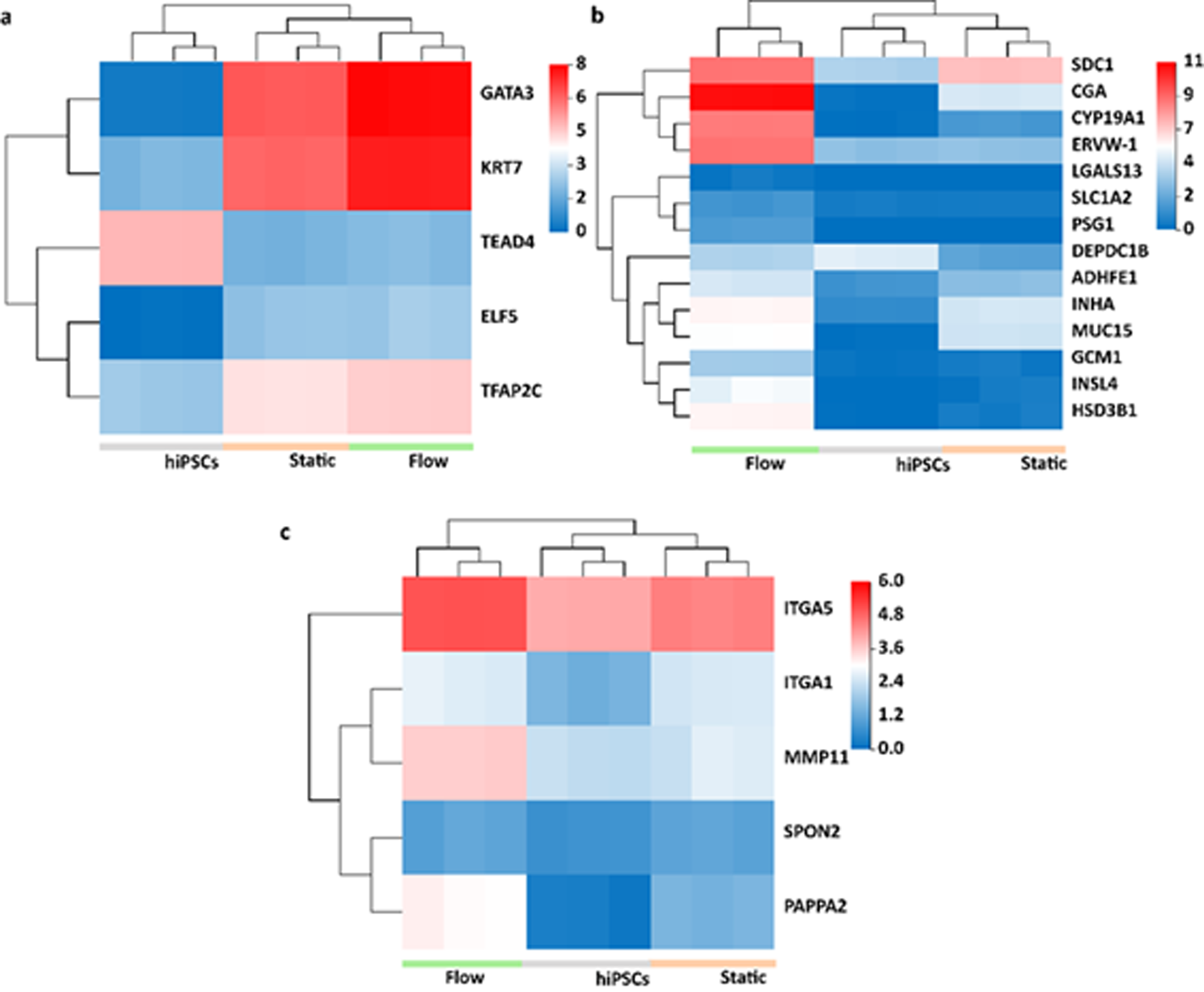

Supplement: Supplementary file 1 [file Image6.tif]

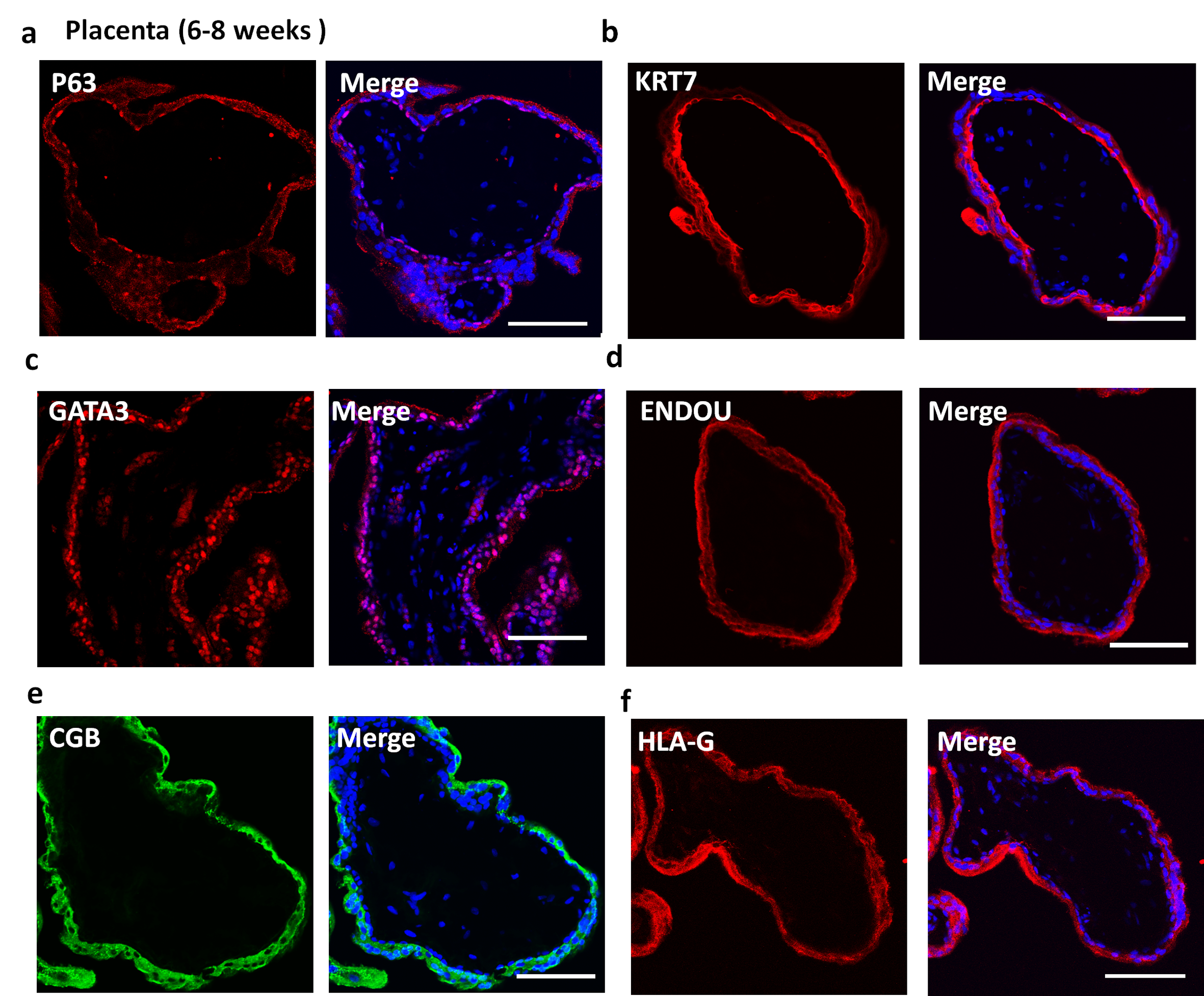

Supplement: Supplementary file 3 [file Image3.tif]

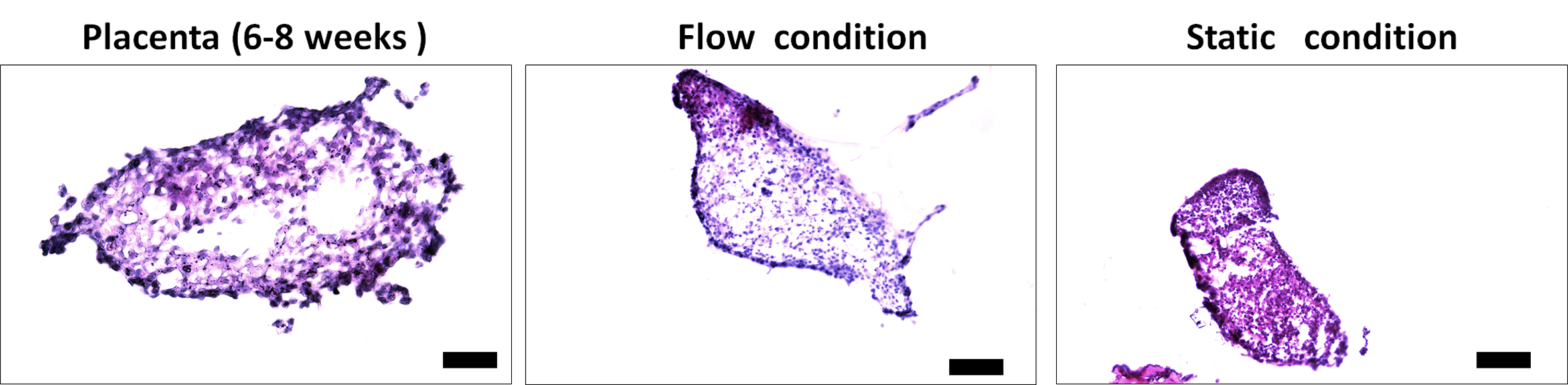

Supplement: Supplementary file 4 [file Image4.tif]

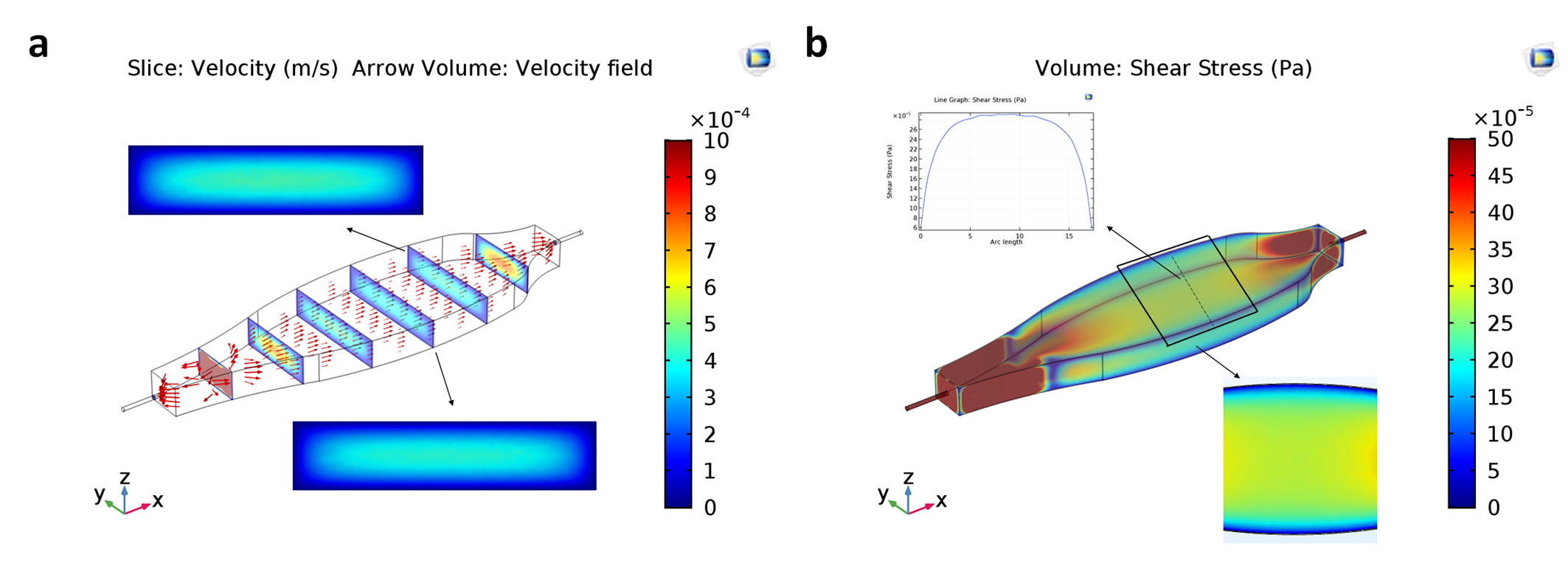

Supplement: Supplementary file 5 [file Image2.tif]

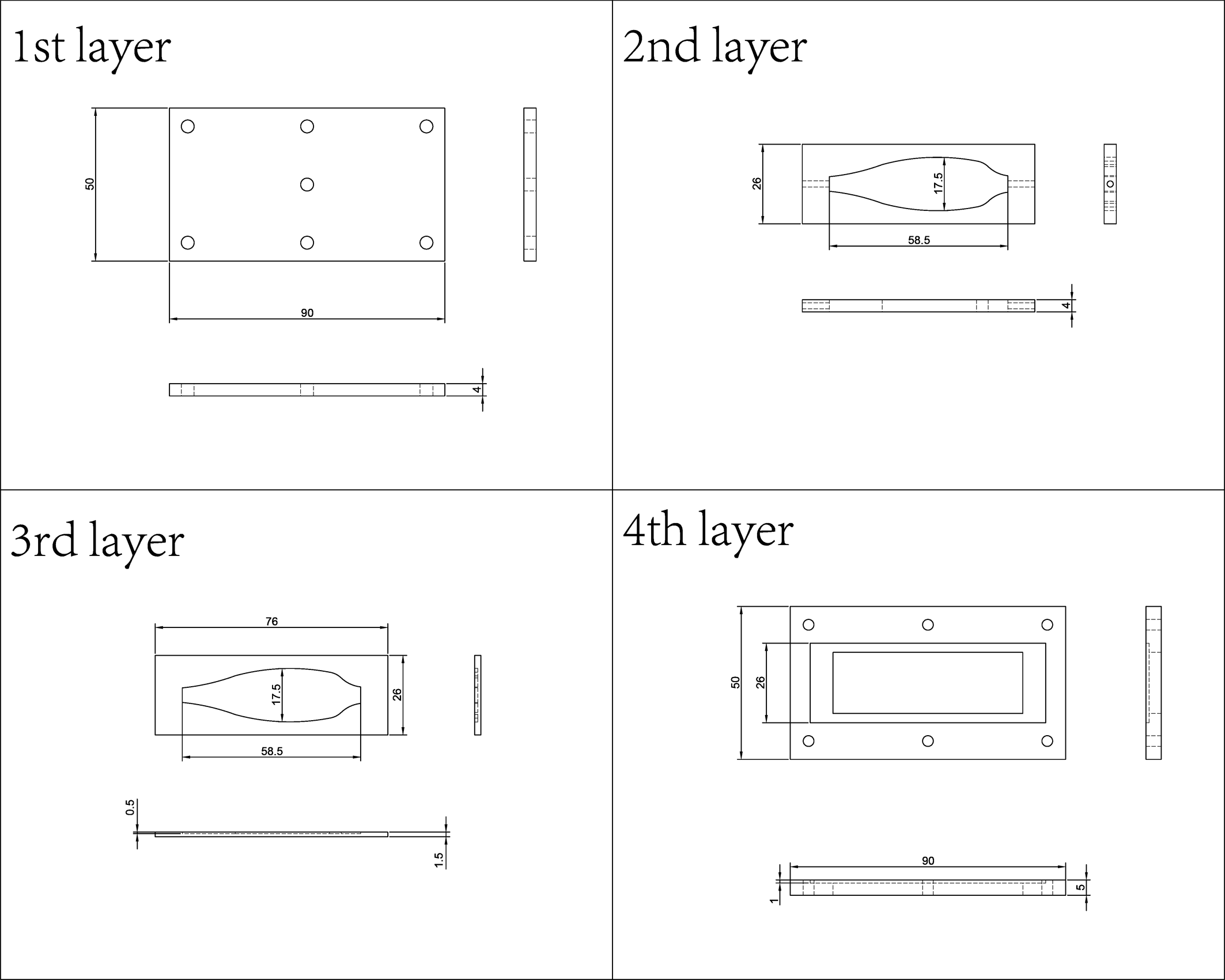

Supplement: Supplementary file 6 [file Image1.tif]

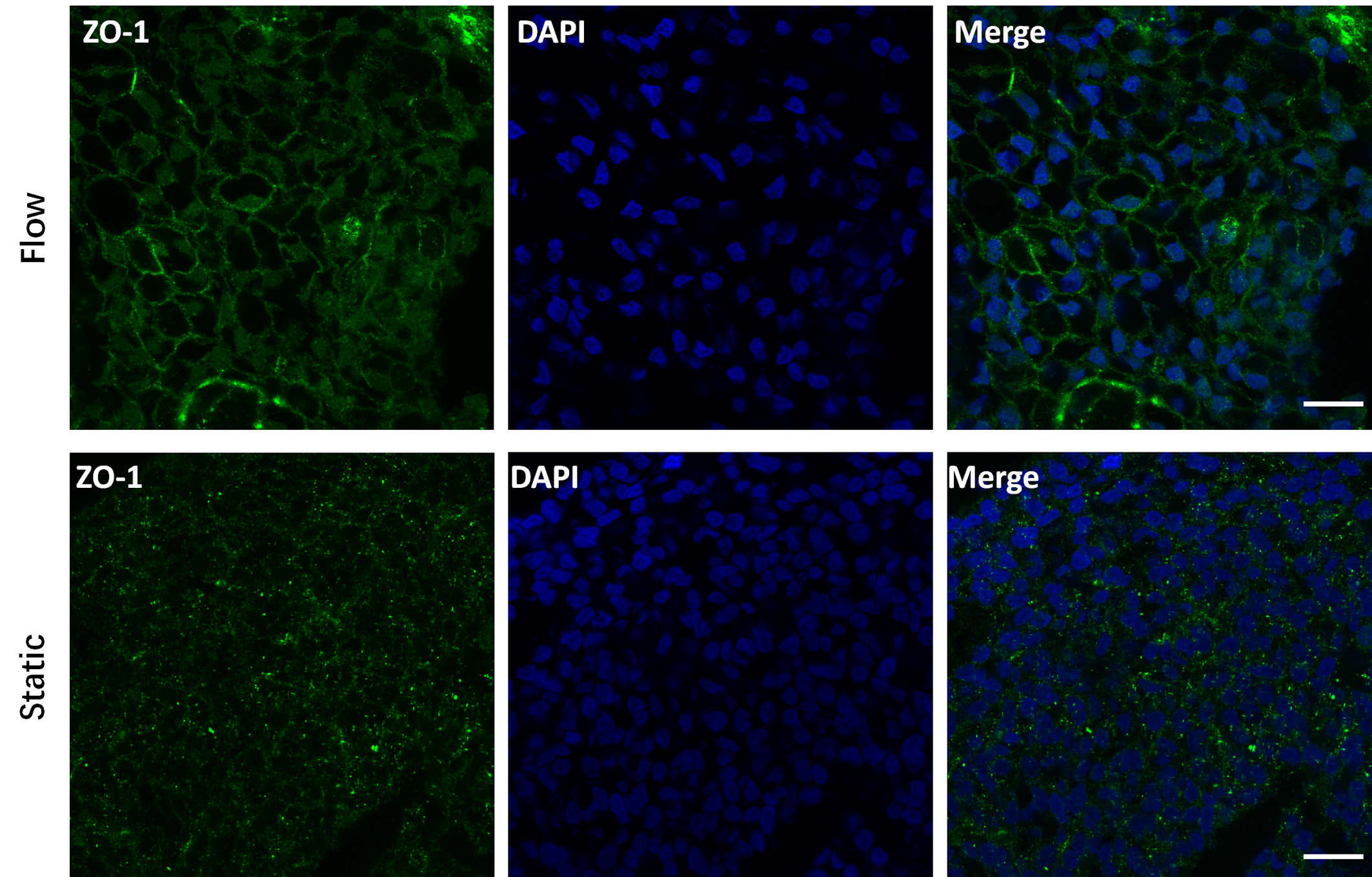

Supplement: Supplementary file 7 [file Image7.tif]

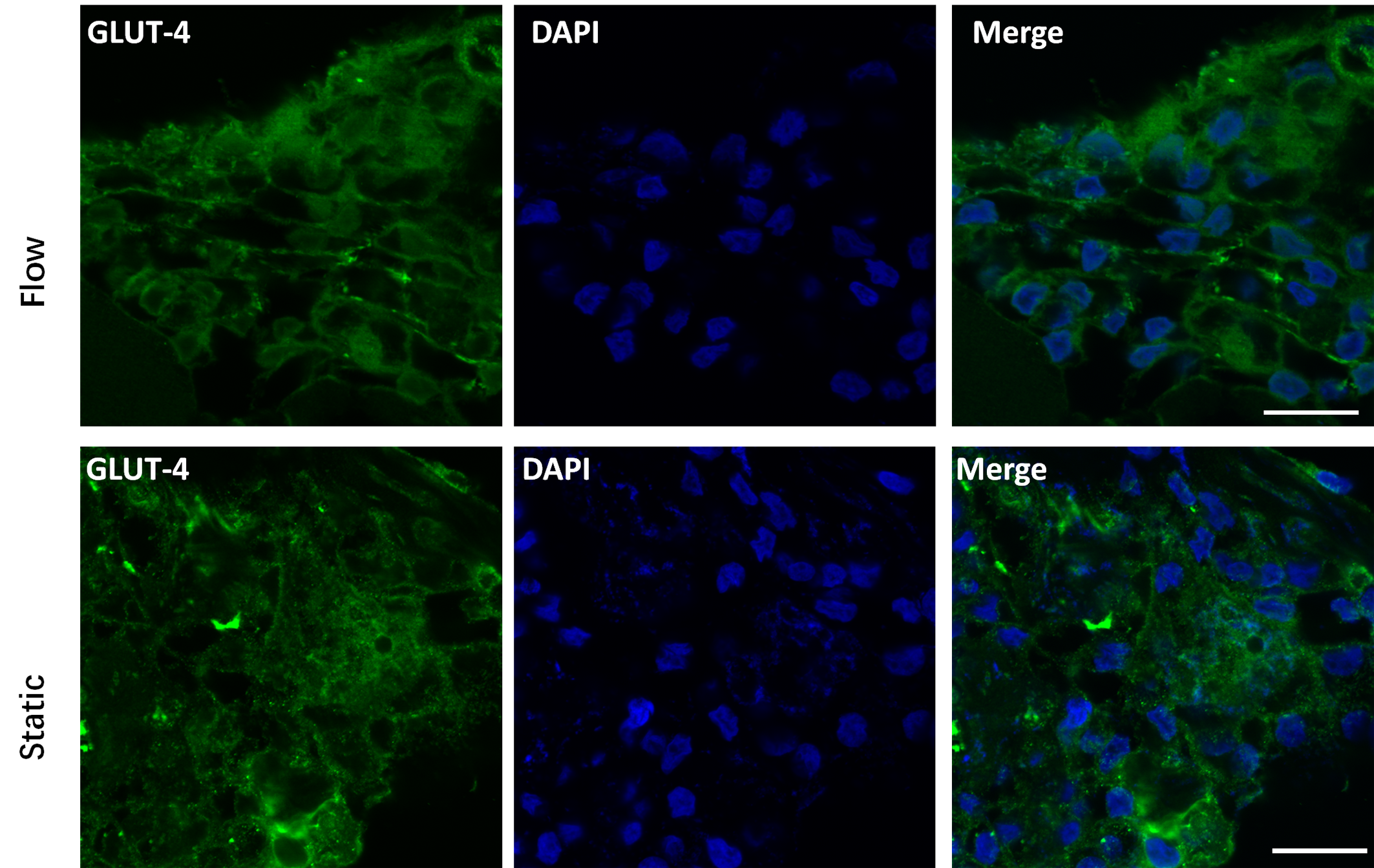

Supplement: Supplementary file 8 [file Image5.tif]
