# Supplementary material for: Fluidic Flow Enhances the Differentiation of Placental Trophoblast-Like 3D Tissue from hiPSCs in a Perfused Macrofluidic Device
Source: Front Bioeng Biotechnol. 2022 Jun 30;10:907104. doi: 10.3389/fbioe.2022.907104 (PMC9280037; doi:10.3389/fbioe.2022.907104)
Supplement: Supplementary file 2 [file Table1.DOCX]

Primer pairs used to examine mRNA expression.

| **Primer name** | **Sequence** |
| --- | --- |
| KRT7 | Fw 5′-TGA ATG ATG AGA TCA ACT TCC TCA G-3′  Rv 5′-TGT CGG AGA TCT GGG ACT GC-3′ |
| P63 | Fw 5′-CTG GAA AAC AAT GCC CAG A-3′  Rv 5′-AGA GAG CAT CGA AGG TGG AG-3′ |
| SDC1 | Fw 5′-CTA TTC CCA CGT CTC CAG AAC C-3′  Rv 5′-GGA CTA CAG CCT CTC CCT CCT T-3′ |
| MMP2 | Fw 5′-GTA TTT GAT GGC ATC GCT CA-3′  Rv 5′-CAT TCC CTG CAA AGA ACA CA-3′ |
| GATA3 | Fw 5′-GCC CCT CAT TAA GCC CAA G-3′  Rv 5′-TTG TGG TGG TCT GAC AGT TCG-3′ |
| Syncytin-2 | Fw 5′-CCT TCA CTA GCA GCC TAC CG-3′  Rv 5′-GCT GTC CCT GGT GTT TCA GT-3′ |
| CGB | Fw 5′-CCC CTT GAC CTG TGA TGA CC-3′  Rv 5′-TAT TGT GGG AGG ATC GGG GT-3′ |
| CGA | Fw 5′-ACA TCC TGC AAA AAG CCC AGA GAA A-3′  Rv 5′-ACT GAA GTA TTG GGG CAC CCG G-3′ |
| HLA-G | Fw 5′-GAG GAG ACA CGG AAC ACC AAG-3′  Rv 5′-GTC GCA GCC AAT CAT CCA CT-3′ |
| GAPDH | Fw 5′-GTG GAC CTG ACC TGC CGT CT-3′  Rv 5′-GGA GGA GTG GGT GTC GCT GT-3′ |
| HSD3B1 | Fw 5′-AGA AGA GCC TCT GGA AAA CAC ATG-3′  Rv 5′-TAA GGC ACA AGT GTA CAG GGT GC-3′ |
| CYP19A1 | Fw 5′-TGC AAA GCA CCC TAA TGT TG-3′  Rv 5′-TTT GTC CCC TTT TTC ACT GG-3′ |
| ITGA5 | Fw 5′-CCC CGA GTA CCT GAT CAA C-3′  Rv 5′-AGG GAT CGA ATG TCT GAG CC-3′ |
| ITGA1 | Fw 5′-CAA ACT GCA GAC CCC ATA TC-3′  Rv 5′-TGA CTT GGC TGA TGT CAG AA-3′ |
| CAML5 | Fw 5′-GGC GGG TGA ACT ACG AG-3′  Rv 5′-GGA GAG TCC CAG CAC AAA AG-3′ |
| CDH5 | Fw 5′-AAA CAC CTC ACT TCC CCA TC-3′  Rv 5′-ACC TTG CCC ACA TAT TCT CC-3′ |
| HOMX1 | Fw 5′-GCC CCA GGA TTT GTC AGA G-3′  Rv 5′-ACA TAG ATG TGG TAC AGG GAG G-3′ |
| GSTA3 | Fw 5′-TGA TGG GAT GAA GTT GGT ACA G-3′  Rv 5′- CAT TCA AAT CTG CCA TAC CTT CTG-3′ |
| FASLG | Fw 5′-TCT TGA GCA GTC AGC AAC AG-3′  Rv 5′-GGG ATA TGG GTA ATT GAA GGG C-3′ |
| FGF8 | Fw 5′-GAC CTA CCA ACT CTA CAG CC-3′  Rv 5′-ACT CGA ACT CTG CTT CCA AAG -3′ |
